# Molecular dynamics simulation analysis of alpha-cobra toxin docked with phytochemical compounds

**DOI:** 10.6026/97320630018834

**Published:** 2022-09-30

**Authors:** Ahmed Mohajja Alshammari, Manal Swaleh Alrashidi, Wasimah Buraikan Alshammari, Bander Hamad Aloufi, Haya Rashed Altamimi

**Affiliations:** 1 Department of Biology, College of Science, University of Hail, Kingdom of Saudi Arabia

**Keywords:** Alpha-cobra toxin, snake venom, scorpion venom, molecular docking, drug targets

## Abstract

It is of interest to document data on the molecular dynamics simulation analysis of alpha-cobratoxin docked with phytochemical compounds. This can be used as effective drug candidates against the snake and scorpion venom. It should be noted experimental verification is needed to further validate the current data.

## Background:

Snake and scorpion venoms contain toxins that impact the prey's circulatory, nerve, muscular, and a variety of other systems [1]. The voltage-gated sodium (Navs) and potassium channels (Kvs) are known targets of scorpion venom peptide toxins, and several of these toxins have antimicrobial action [2]. For example, compounds with anticoagulant and antiviral properties were identified in scorpion venoms or synthesized from venom gland cDNA libraries. The toxin from snake bites and scorpion venom are studied with different chemical inhibitors [3]. Sequence homology help in structure based drug design [4]. The toxin is alpha-Cobratoxin, which prevents nicotinic acetylcholine receptors 7/CHRNA7 from working in muscles and neurons [5]. The tropical and subtropical regions of Africa and Asia are home to the cobras that are responsible for the production of these toxin [6]. These snakes secrete venom that is a combination of several compounds, including proteins, carbohydrates, and other chemicals [7]. Muscle-type and neuronal-type nAChRs are susceptible to the antagonistic and slowly reversible binding of beta-cobratoxin [8]. The cobratoxin from Thailand's cobra is known [9]. Muscle paralysis results from a bite from a Thailand Cobra [10]. The venom will induce a loss of strength due to a blockage in nerve communication. An eye movement abnormality and palpebral ptosis (drooping of the eyelids) are two of the early indications of paralysis [11]. A diverse variety of compounds and phytochemicals are produced by plants [12]. These compounds includes flavonoids, lignin’s, tannins, phytoalexins, and polyphenols [13]. Flavonoids and polyphenols are the source of secondary metabolites and rich in nuts, spices, fruits, seeds, tea, red wine, and vegetables stems that are consumed by people in excess amount [14, 15]. Biotic and abiotic stress in plants responsible for the production of those compounds in the presence of UV light, insects, and pathogens [15, 16]. Computational drug designing has become a cost and time effective approach to design the new drugs [17]. Therefore, it is of interest to document the molecular dynamics simulation analysis data of alpha-cobratoxin docked with phytochemical compounds.

## Methodology:

### Structure retrieval and library preparation:

The toxin that is present in both snake bite and scorpion venom was alpha-cobratoxin. The PDB structure for this toxin was retrieved from the Protein Database with the PDB ID 6ZFM [18]. The protein was found in the bounded state with a peptide inhibitor. The protein structure was pre-processed using the MOE software. The peptide inhibitor was removed. Out of four chains of toxins, one was selected. The chain was energy minimized using the Amber99 forcefield in MOE software to remove all constraints from the pre-processing and stability of the toxin for further processing [19]. A library of 500 phytochemicals was built to screen against the targeted protein. After virtual screening, five ligands were selected with their inhibition properties against venom, identified. Compounds such as vidalenolone [20], edunol [21], elaidamide [22], neostigmine [23] and a synthetic compound like Ethyl 2-((4-chlorobenzoyl)thio)acetate [24] are known to have anti-venom properties. Both protein and ligand were preprocessed in the MOE software, where energy minimization was done for alpha-Cobratoxin and ligands using the Amber99 and MMFF9x force field, respectively. The excessive chains from the alpha-cobratoxin were removed as a part of preprocessing.

### Molecular docking Protocol:

Molecular docking was performed by using Gold suite 5.3.0 using the standard operating procedures [25]. The higher the gold fitness score, the higher the ligand has to inhibit interacting properties [26]. After docking, the interacting pattern was analyzed and preceded for molecular dynamic simulation.

### Molecular dynamic Simulation:

Molecular dynamic simulations were performed to check the stability of the bonds that have been made during docking protocol. To understand the stability of the docked complexes generated from docking protocol, 50 ns of Molecular Dynamics (MD) simulation was performed by using software known as SCHRODINGER [27, 28]. The stability of the complexes was evaluated by CαRMSD [29].

### Physiochemical property profile & toxicity prediction:

All the selected molecules were analyzed through the Molinspiration server for Lipinski’s Rule of Five. This rule explains the different drugs properties like absorption, metabolism and drug secretion in the human body. Compounds are evaluated on the basis of this rule as it includes different values like Molecular weight fewer than 500 Daltons, H-bound donor less than five, H-bound acceptors less than ten and log P-values must be <5 [30]. Swiss-ADME software was used to predict the pharmacokinetic properties like (absorption, metabolism, distribution, excretion and toxicity) [31].

## Results:

Virtual screening of compounds: After successful virtual screening of 500 compounds top 5 inhibitors were chosen for further study against the target protein alpha-cobratoxin 2D visualization of those compounds were mentioned in the Figure 1(see PDF).

## Binding pocket identification:

The peptide that was removed from the PDB structure of alpha-Cobratoxin gave information about the binding pocket of the alpha-Cobratoxin. The binding pocket residue constituted Tyr-2, Met-3, Trp-4, Asp-5, Gly-6, Pro-7, Ile-32, Arg-33, Gly-34, Lys-35, Arg-36 Val-37, Asp-38, and Leu-39 [18]. These residues were utilized in further steps for the optimum attachment of ligand within the interacting domain.

## Molecular docking:

Then the energy minimized structure and toxin_inhibitors database was undergone through molecular docking protocol by utilizing GOLD suite software. The GA algorithm was selected for gold fitness score, and the 50 iterations were performed. The resultant docked complexes were observed for the interacting patterns and hydrogen bond interactions with respect to their respective gold fitness score (Table 1 - see PDF)

During interaction analysis, all ligands bound within the binding domain, validating the inhibitory domain of toxin. Leu-39 observed strong hydrogen bonds with all inhibitors, stating the importance of this residue in this interaction, thus inhibition. Edunol complex with a gold fitness score of 50 showed the maximum interacting pattern with three hydrogen bonds and two hydrophobic interactions. This pattern showed that inhibitor2 represents induced-fit properties within the binding domain. First (Vidalenolone) and fourth (Ethyl,2-((4-chlorobenzoyl)thio)acetate) complex (gold fitness score 47) (gold fitness score 42 and 47) showed three hydrogen bonds and one hydrophobic interaction. Neostigmine complex exhibited two hydrogen bonds and one hydrophobic interaction with a gold fitness score of 43. The change in interaction pattern is due to a change in the cationic nature of the ligand, which forms inter-polar interaction within the ligand. Elaidamide complex was declared as the least active compound with a gold fitness score of 27 and only one interaction. 3d visualization of the docked complexes was indicated in the Figure 2(see PDF).

A correlation plot was made to find the relation of the interaction score (gold score) and the inhibitor's respective molecular weight. The correlation plot for the complexes was made with a gold fitness score on the y-axis and molecular weight on the x-axis. The graphs represent the interaction that the inhibitors have shown is due to their molecular weight. The R-value of the correlation plot was 0.77, which shows a strong positive correlation as well (Figure 3 - see PDF). A ligand shows its interaction for two reasons: its activity and its transport property. The transport property is the ability of a ligand to diffuse in the membrane for its way towards the target. As the cell membrane is hydopobic in nature, it allows hydrophobic molecules to pass through. The more the compound's molecular weight, the more it will carry hydrophobic properties, thus interacting easily and efficiently with the target. Here the Edunolcomplex with a gold fitness score of 50 showed highly active behavior. Vidalenolone,Neostigmine, andEthyl,2-((4-chlorobenzoyl)thio)acetatecomplexes make a group of similar behavior and interacting pattern (as mentioned earlier). Elaidamide, however, showed an outlier or least active behavior due to the polar nature of the structure.

## MD simulation:

For this purpose, SCHRODINGER software was utilized. The system was built by TIP3P water molecules. The ions were added as sodium or chloride. After taking the system towards equilibrium, the MD simulations were run for 100nsec at the specific temperature and pressure. After the simulations, the trajectory was analyzed by CαRMSD, declaring the stability of complexes and interaction during the simulation time (Table 2 - see PDF). During the simulation, it was observed that some bonds remained consistent throughout the simulation, like Leu-39, Val-37, which were stable in the docking studies as well. The purpose of the MD simulation is to check the compound's high stability to some extent. Some bonds disappeared with the evolution of time.

As observed from Table 2(see PDF), most residues remain stable over time. To observe the simulation trajectory, the RMSD plot was observed (Figure 4 - see PDF). The RMSD plot gave the snapshot of the simulation with time [32]. Here the most stable pattern was observed for Edunol complex, which showed RMSD of 3.2 Å for the toxin and 12 Å for the respected inhibitor. The complex represented the highly fluctuating pattern till 90nsec while it got stable towards the end. For Ethyl,2-((4-chlorobenzoyl)thio)acetate, RMSD was observed 9 Å and 28 Å for toxin and inhibitor, respectively. The complex was fluctuating at the start; however, both ligand and protein stabilized at the middle without overlapping. This indicated that the structures were stable but lost some interacting pattern [33]. Vidalenolone complex showed the RMSD of 12 Å and 8 Å for toxin and inhibitor, fluctuating throughout the period. This behavior suggested the minimum stability achieved by this compound. Neostigmine complex also exhibited fluctuation behaviour within 100nsec with RMSD of 7 Å and 36 Å for toxin and inhibitor, respectively, suggesting the minimum stability in an interacting pattern. Lastly, the Elaidamide complex showed maximum fluctuation during the simulation period with a high RMSD value of 14 Å for toxin and 40 Å for the inhibitor.

## Drug likeness and ADMET profiling:

Utilizing the molinspration service, a drug scanning was done to evaluate the drug-like qualities of the top Compounds. The Lipinski five-rule is now accepted as the norm. This rule emphasises key aspects of drug metabolism, interactions, and excretion in the human body, including pharmacokinetics. Lipinski's five conditions were all met by some compounds, and they also had drug-like characteristics including molecular weight (Table 4 - see PDF). Several pharmacokinetic factors were assessed using ADME and AdmetSAR. Pharmacokinetic traits can be used to assess the ADME and toxic effects of the top candidate agents. The ADMET characteristics of derived phytochemicals for both targets are shown in Table 5(see PDF). Many drugs do not utilise this pathway in their development due to cytotoxicity and poor pharmacokinetic qualities. To find active lead components during the early stages of drug discovery, high-performance and rapid ADMET profiling research is prioritized. By analysing their ADMET profiles, promising compounds' drug-likeness was also confirmed using the ADMET Lab (Table 4 - see PDF).

## Discussion:

Computer aided compound library is available for developments in chemo informatics compounds [34]. Hence, for designing and screening innovative compounds molecular docking seems to be a valuable technique against the appalling diseases [35, 36]. The present study illustrated that by identifying the active sites of the target protein (Alpha-Cobra Toxin), we can inhibit its expression. There are some compounds those have considerable interactions with the target protein involved in Alpha-Cobra Toxin. Molecular properties and drug-likeliness of the selected complexes were estimated according to the "Lipinski Rule of Five". This rule states that, the molecular weight of the compound must less than 500 Daltons, less than 5 Hydrogen bond donors, no more than 10 Hydrogen bond acceptors, and a logP value fewer than 5. All compounds fulfill the Lipinski's Rule of Five and show no violation. Selected compounds have low scoring values as compared to the standard drugs and have RMSD values less than 3. ADMET analysis is a challenging process in the drug discovery. This is achieved through Swiss ADME database and showed that selected compounds have good pharmacokinetic properties. Drug development process of many drugs do not go through the process just because of the poor pharmacokinetic properties and toxicity [37]. Identification of active lead compounds depends upon the High-performance and fast ADMET profiling assays at early drug discovery [38]. ADMET profiling shows that there is no side effect of absorption of all potential compounds. The associated ADMET properties of potential compounds for different models such as P-glycoprotein substrates, BBB penetration, and gastrointestinal absorption showed positive results that strongly support compounds' ability to function as a drug candidate. Cytochrome P450 (CYP) is a cluster of isozymes comprising fatty acids, bile acids, carcinogens, steroids, and the metabolism of drugs. Fifty-seven CYPs are encoded by human genome, of which fifteen are participating in the xenobiotic chemicals and another drug metabolism [39]. CYP enzymes association is very important for drug metabolism almost 75 percent of the phase 1 of drug metabolism depends upon its association [40].

## Conclusions:

Data shows that phytochemicals (vidalenolone, edunol, neostigmine, ethyl,2-((4-chlorobenzoyl)thio)acetate and elaidamide) binds to Alpha-Cobra toxin involved in snake and scorpion. This can be used as effective drug candidates against the snake and scorpion venom. It should be noted experimental verification is needed to further validate the current data.
